# Supplementary material for: ROCK regulates the intermittent mode of interstitial T cell migration in inflamed lungs
Source: Nat Commun. 2017 Oct 18;8:1010. doi: 10.1038/s41467-017-01032-2 (PMC5647329; doi:10.1038/s41467-017-01032-2)
Supplement: Supplementary file 1 — Supplementary Information [file 41467_2017_1032_MOESM1_ESM.pdf]

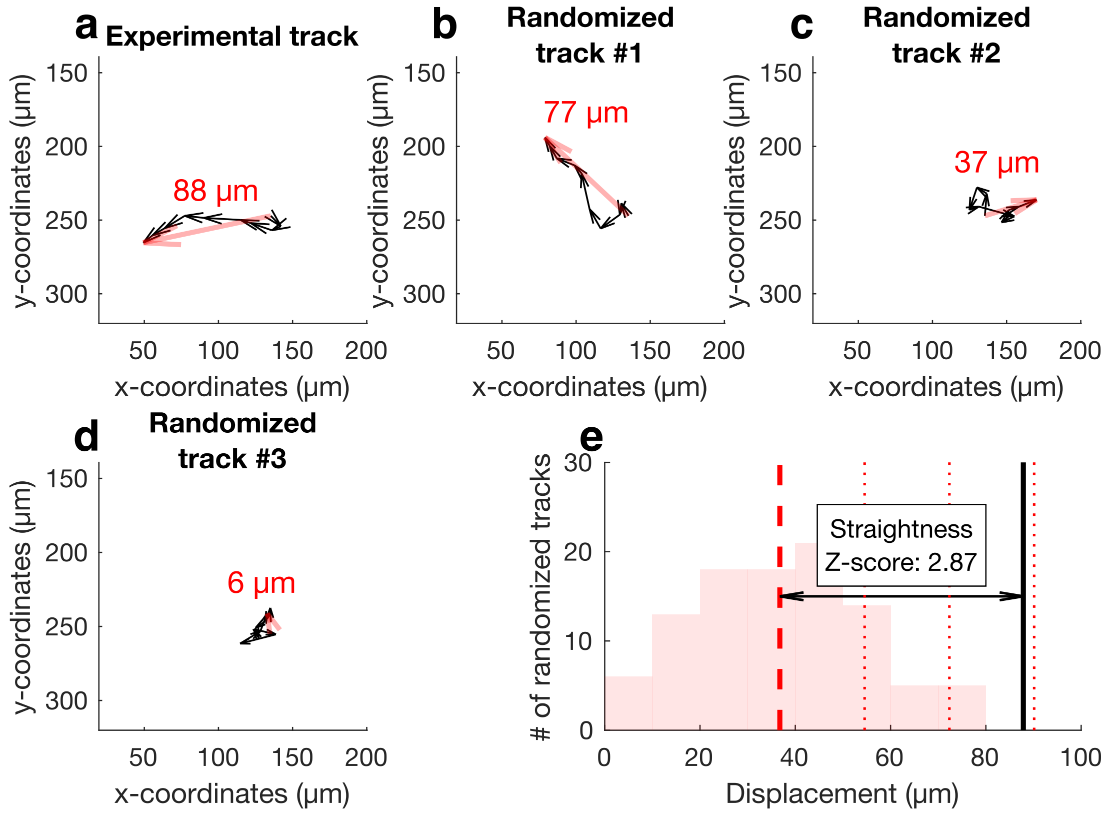

Supplementary Figure 1. Generation of randomized tracks and computation of Straightness Z-score

(a-d) Tracks are depicted as a series of velocity vectors (black arrows). The distance between the beginning and the end of each track is depicted graphically (red vector) and numerically (red numbers).

(a) Shown is a straight experimental track.

(b-d) 100 randomized track-derivatives were generated by randomization of the order and orientation of their vectors. The track derivatives with the highest (b), median (c) and lowest (d) displacement are shown.

(e) Histogram of the displacements of all randomized track derivatives. The red dashed line represents the mean displacement. The three dotted lines represent displacements that exceed the mean by one, two and three standard deviations. The solid black line represents the displacement of the original experimental track. The arrow indicates how many standard deviations the actual displacement differs from the mean displacement of the randomized population ("Straightness Z-score").

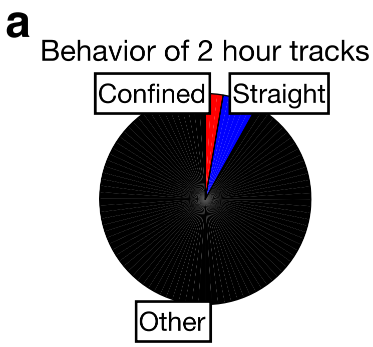

Supplementary Figure 2. Analysis of confined and straight migration in randomized tracks

GFP+ CD8+ effector T cells were injected i.v. into LPS-treated mice. After two to five days, lungs were explanted and imaged with two-photon microscopy.

(a) Estimation of background noise level of switching between confined and straight migration in 2 hour tracks (n= 37). Analyzed track children were randomized before computation of the straightness Z-score (see Methods for a more detailed description of the approach).

The analysis was performed on pooled data obtained from 3 independent experiments.

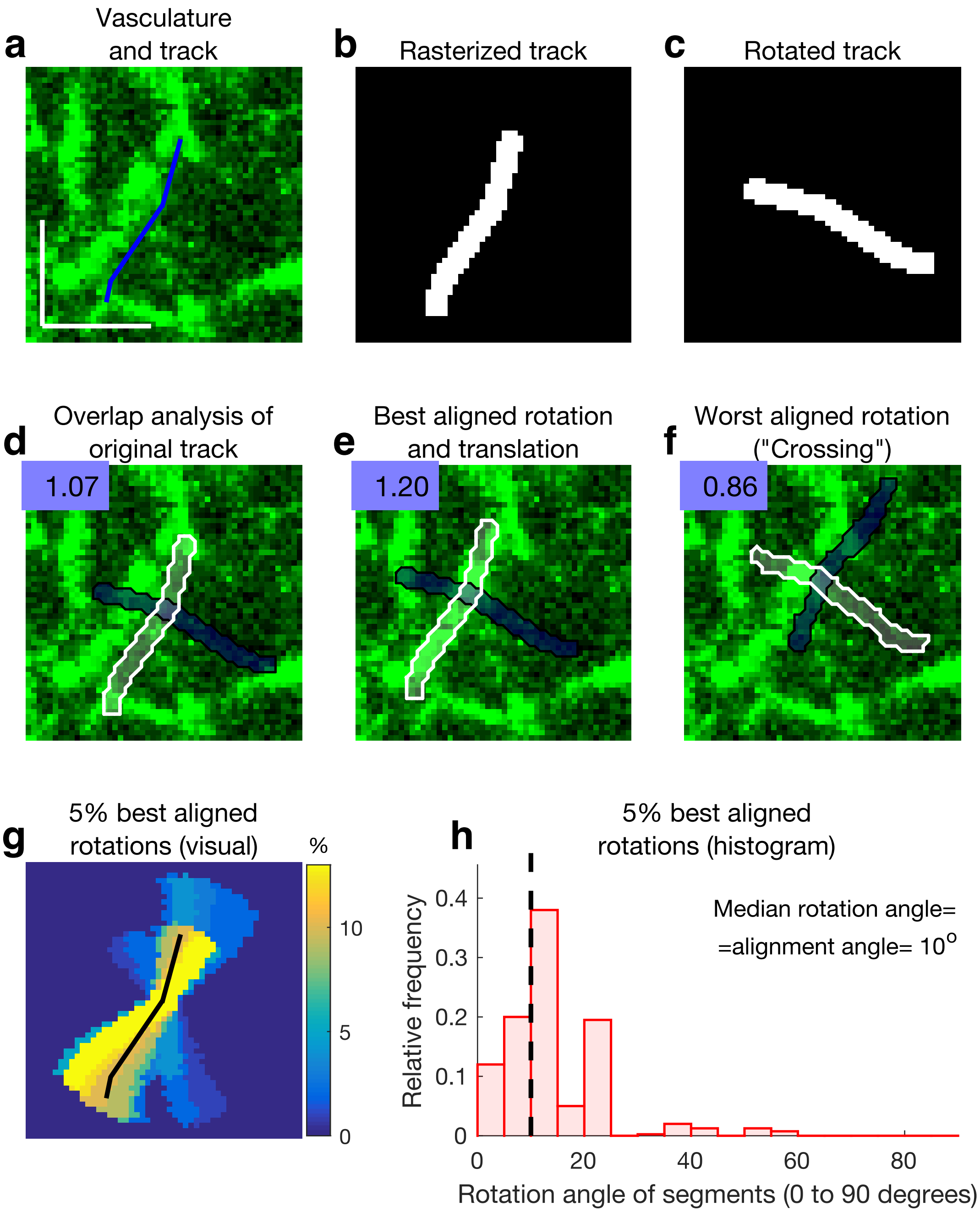

28

29 **Supplementary Figure 3. Computational approach to quantify alignment between**
30 **tracks and vasculature**

31 (a-c) Rasterization, rotation and translation of track segments.

(a) Tracks were split into segments with a maximum displacement of 24 µm. Shown is a representative segment (blue) superimposed on the intensity map of the vasculature stain (green). Scale bar= 15 µm.

(b) Segment is converted from vector format into a raster image.

(c) Rotation (from 0 to 355 degrees every 5 degrees) and translation (-5 to 5 pixels horizontally and vertically) of segment images leads to a total of 8712 "segment derivatives". Shown is a segment derivative rotated by 90 degrees (no translation).

(d-f) Detection of segments that are best aligned with the vasculature ("Overlap analysis").

For each "given" segment (white area and edge) and its "perpendicular" segment (blue area and black edge) the median intensity of overlapping vasculature pixels (green) was computed. The "overlap ratio" between overlap indices of "given" and "perpendicular" segment images is shown at the top left of each panel.

Depiction of overlap analysis for the "original" segment (e), and for a well-aligned (e) and a poorly aligned (f) segment derivative.

(g, h) Computation of the "alignment angle" between segments and the vasculature.

All segment derivatives were ranked according their "overlap ratio" and the 5% segments with the highest overlap ratio, i.e. those that were best aligned with the vasculature, were isolated for further analysis.

(g) Visual depiction of the frequency of the rotations of the best-aligned segment derivate images (colored pixels) and the original segment vector (black line).

(h) Histogram of the rotation angles between the original segment and 5% best aligned segment derivatives (range 0 to 90 degrees). The alignment angle is defined as the median rotation angle (indicated by the dashed line).

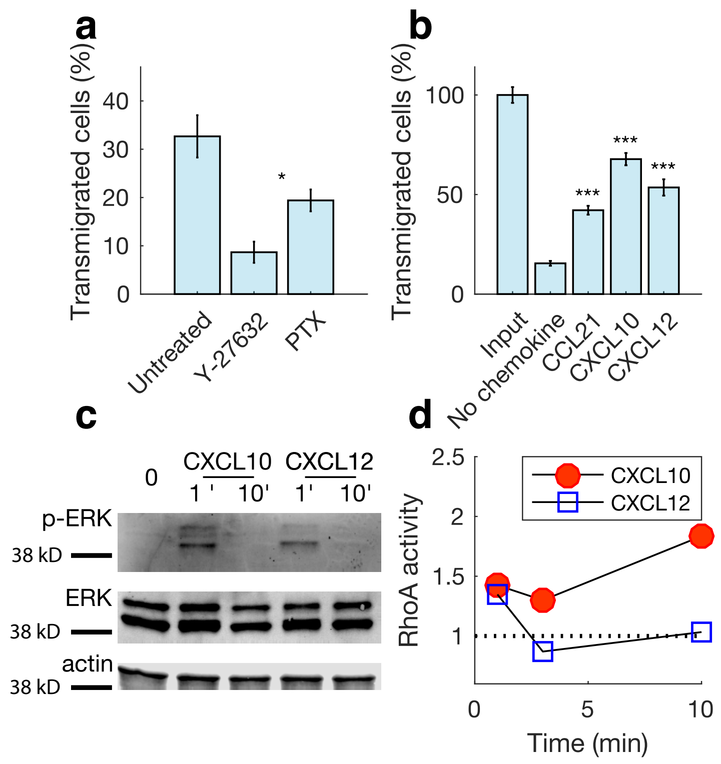

Supplementary Figure 4. *In vitro* analysis of ROCK- and chemokine-signaling in CTL

The response of *in vitro* generated cytotoxic T lymphocytes (CTL) to chemokines and pharmacological inhibitors was analyzed.

(a) Transwell analysis of CTL without addition of exogenous chemokine. Cells pretreated with pertussis toxin (PTX; 2 hours) or Y-27632 (10 minutes) before adding the cells into the transwell inserts. The inhibitors were also present during the transwell assay. The pore size of the transwell inserts was 3 µm. P-values for transmigration in the presence of Y-27632 versus PTX were computed with the Mann-Whitney assay (*: $p<0.05$).

(b) Chemotaxis towards CCL21, CXCL10 and CXCL12 was analyzed with transwell assays. Three independent experiments were pooled (n= 9 wells). P-values for transmigration in the presence of chemokines versus no chemokine were computed with the Mann-Whitney assay (***: $p<0.0001$). The pore size of the transwell inserts was 5 µm.

(c, d) Lysates from unstimulated or chemokine-stimulated CTL were analyzed. Protein levels of actin, ERK1/2 and phosphrylated ERK1/2 were determined with Western blot analysis (b). Levels of active RhoA were determined with a "RhoA activity kit" (c).

Dashed line indicates baseline activity in the absence of stimulation. Experiments were repeated three times (b, c). One representative experiment is shown.

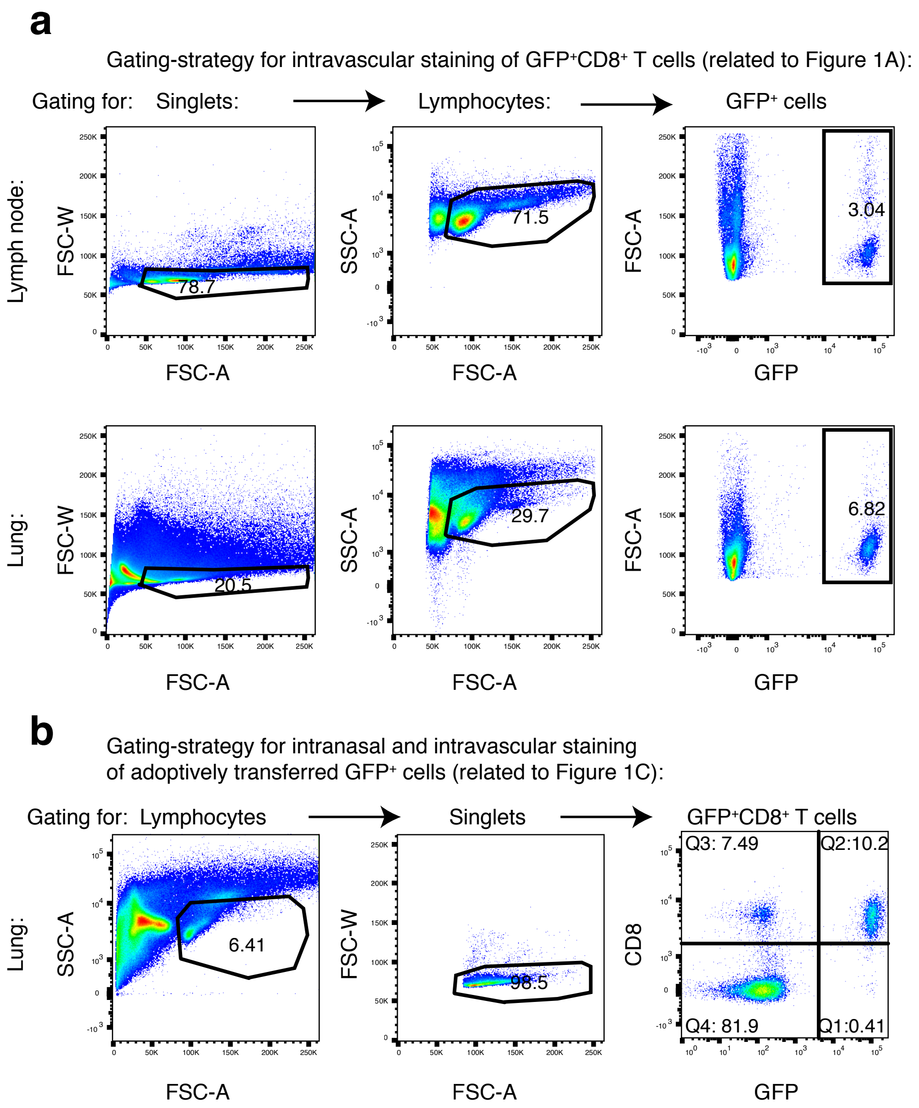

Supplementary Figure 5. Gating strategy for flow cytometry analysis

(a, b) Single cell suspensions obtained from lymph nodes or lungs were analyzed with flow cytometry. Cells were gated using forward scatter (FSC) and side scatter (SSC) values to enrich for lymphocytes. Gating on FSC-W versus FSC-A was used to remove doublets and maintain events corresponding to single cells.

(a) A GFP versus FSC-A gate was used to identify GFP$^+$ (adoptively transferred) cells. Cells from this gate were used for the analysis in Fig. 1a.

(b) A GFP versus CD8 gate was used to identify adoptively transferred GFP$^+$CD8$^+$ T cells. Cells from this gate were used for the analysis shown in Fig. 1c.

Supplementary Table 1. Statistical analysis of curve-fitting for instantaneous speeds

'Distribution'	'AICc'	'Neg. Log Likelihood'	'KS'
'Lognormal'	-8268	-4136	1
'Power Law'	-5326	-2664	1
'Rayleigh'	-6757	-3379	1

To quantify the best fits of the tested distributions, we calculated AICc, negative log likelihood and performed the Kolmogorov-Smirnov test (KS). The best fits were achieved with mu= -3.313 and sigma= 0.997 for the lognormal distribution; with an exponent mu= 1.336 for the power-law distribution; with the scale parameter sigma= 0.053 for the Rayleigh distribution. For the AICc and negative likelihood measurements, more negative values are indicative of better fits. For the KS test, unity indicates rejection of the null hypothesis, i.e. the distribution is inconsistent with empirical data.
