# Supplementary material for: ROCK regulates the intermittent mode of interstitial T cell migration in inflamed lungs
Source: Nat Commun. 2017 Oct 18;8:1010. doi: 10.1038/s41467-017-01032-2 (PMC5647329; doi:10.1038/s41467-017-01032-2)
Supplement: Supplementary file 3 — Description of Additional Supplementary Files [file 41467_2017_1032_MOESM3_ESM.pdf]

Description of Additional Supplementary Files

File Name: Supplementary Movie 1
Description: Migration of CD8+ effector T cells in explanted lungs Left panel: Maximum projection of movie-sequence capturing adoptively transferred T cells (green) within an explanted lung. Trajectories (white lines) show the position of analyzed cells over time. Hours:minutes:seconds are shown in the left bottom corner. Right panel: 3D depiction of cell positions (green circles) and trajectories (blue lines) over time. To improve depth perception, the image volume is rotating during replay.

File Name: Supplementary Movie 2
Description: Migration of a single CD8+ effector T cell that switches between slow and fast migration Left panel: Maximum projection of movie-sequence capturing adoptively transferred T cell (green) within an explanted lung. The trajectory (white lines) shows the position of the analyzed cell over time. Hours:minutes:seconds are shown in the left bottom corner. Right panel: 3D depiction of cell position (green circle) and trajectory (blue lines) over time. To improve depth perception, the image volume is rotating during replay.

File Name: Supplementary Movie 3
Description: Migration of a single CD8+ effector T cell that switches between slow, confined and straight migration Left panel: Maximum projection of movie-sequence capturing an adoptively transferred T cell (green) within an explanted lung. The trajectory (white lines) shows the position of the analyzed cell over time. Hours:minutes:seconds are shown in the left bottom corner. Right panel: 3D depiction of cell position (green circle) and trajectory (blue lines) over time. To improve depth perception, the image volume is rotating during replay.

File Name: Supplementary Movie 4
Description: Migration of a motile T cell captured by intravital imaging Left panel: Maximum projection of an adoptively transferred T cell (green) captured by intravital two-photon imaging. The trajectory (white lines) shows the position of the analyzed cell over time. Hours:minutes:seconds are shown in the left bottom corner. Right panel: 3D depiction of cell position (green circle) and trajectory (blue lines) over time. To improve depth perception, the image volume is rotating during replay.

File Name: Supplementary Movies 5-7
Description: T cell movement in relation to lung-associated vasculature Left panels: Maximum projection of movie-sequences capturing T cells (green) within explanted lungs that also contain a lectin-stain of the vasculature (red). Hours:minutes:seconds are shown in the left bottom corner. Right panel: 3D depiction of cell position (green circle) and trajectory (blue lines) over time. To improve depth perception, the image volume is rotating during replay.

File Name: Supplementary Movie 8
Description: Distinct T cell tracks follow similar paths Shown are T cells (in green) and lung vasculature (in red). White lines indicate the tracks of two distinct T cells that follow similar paths. The track of cell 1 turns blue and the track of cell 2 turns green during motility.

File Name: Supplementary Movie 9
Description: T cell movement before and after treatment with pertussis toxin Shown are T cells before (left two panels) and two hours after treatment with pertussis toxin (PTX; right two panels) in the same field. Far left and center right: Maximum projection of movie showing migration of T cells (green) and tracks of randomly selected T cells (white lines). Hours:minutes:seconds are shown in the left bottom corner. Center left and far right: 3D depiction of cell positions (green circles) and trajectories (blue lines) over time. To improve depth perception, the image volume is rotating during replay.

File Name: Supplementary Movie 10
Description: T cell movement before and after treatment with Y-27632 Shown are T cells before (left two panels) and after treatment with Y-27632 (right two panels) in the same field. Far left and center right: Maximum projection of movie showing migration of T cells (green) and tracks of randomly selected T cells (white lines). Hours:minutes:seconds are shown in the left bottom corner. Center left and far right: 3D depiction of cell positions (green circles) and trajectories (blue lines) over time. To improve depth perception, the image volume is rotating during replay.

File Name: Supplementary Movie 11 and 12
Description: Modeling of interactions between T cells and target cells Movies show T cell movement resulting from intermittent (left panels), confined (middle panels) and straight (right panels) migration models. T cells are represented by green boxes and target cells are represented by red boxes. Days:hours:minutes:seconds are shown in the left bottom corner of each panel. Supplementary movie 11 shows a snapshot the T cells at each shown time frame. Supplementary movie 12 shows the cumulative area that is covered by all T cells.
